# Pathogenesis of bone disease in multiple myeloma: from bench to bedside

**DOI:** 10.1038/s41408-017-0037-4

**Published:** 2018-01-12

**Authors:** Evangelos Terpos, Ioannis Ntanasis-Stathopoulos, Maria Gavriatopoulou, Meletios A. Dimopoulos

**Affiliations:** Department of Clinical Therapeutics, National and Kapodistrian University of Athens, School of Medicine, Athens, Greece

## Abstract

Osteolytic bone disease is the hallmark of multiple myeloma, which deteriorates the quality of life of myeloma patients, and it affects dramatically their morbidity and mortality. The basis of the pathogenesis of myeloma-related bone disease is the uncoupling of the bone-remodeling process. The interaction between myeloma cells and the bone microenvironment ultimately leads to the activation of osteoclasts and suppression of osteoblasts, resulting in bone loss. Several intracellular and intercellular signaling cascades, including RANK/RANKL/OPG, Notch, Wnt, and numerous chemokines and interleukins are implicated in this complex process. During the last years, osteocytes have emerged as key regulators of bone loss in myeloma through direct interactions with the myeloma cells. The myeloma-induced crosstalk among the molecular pathways establishes a positive feedback that sustains myeloma cell survival and continuous bone destruction, even when a plateau phase of the disease has been achieved. Targeted therapies, based on the better knowledge of the biology, constitute a promising approach in the management of myeloma-related bone disease and several novel agents are currently under investigation. Herein, we provide an insight into the underlying pathogenesis of bone disease and discuss possible directions for future studies.

## Introduction

Multiple myeloma (MM) is a plasma cell dyscrasia characterized by malignant proliferation of monoclonal plasma cells in the bone marrow. MM-induced bone disease is a hallmark of MM; up to 80% of patients present with osteolytic bone lesions at diagnosis and have an increased risk of skeletal-related events (SREs) associated with increased morbidity and mortality^1^. Approximately 60% of myeloma patients will develop a fracture during the disease course^2^. The therapeutic strategy of MM-induced bone disease includes a multimodality approach ranging from bisphosphonates and targeted therapies to local irradiation and orthopedic intervention^1^. Currently, bisphosphonates remain the gold standard treatment for myeloma bone disease^1^; they both inhibit osteoclasts and induce MM cell apoptosis, while they exert an immunomodulatory effect on bone microenvironment^3^. Zolendronic acid is combined with novel anti-myeloma agents and reduces SREs, improves quality of life, while it prolongs both disease-free and overall survival at least in subsets of MM patients. However, adverse effects such as renal impairment and jaw osteonecrosis, as well as the unmet need of reversing bone destruction necessitate the development of novel agents^3^. Among those that are currently under investigation, denosumab, a RANKL inhibitor, has demonstrated promising results^4^. The cardinal events in the pathogenesis of bone disease in MM are the increased osteoclast activity in combination with osteoblast inhibition^5^. These aspects are regulated by numerous signaling pathways. Understanding of the underlying pathogenetic mechanisms of bone destruction is crucial for the effective management and the improvement of quality of life of MM patients. Thus, the aim of this review is to provide a clear insight into the underlying pathogenesis of bone disease in MM patients.

## An overview of the cellular approach of MM-related bone disease

### Osteoclasts and osteoblasts in normal bone metabolism

Bone remodeling constitutes a dynamic lifelong process in adults that is vital for the skeleton in order to sustain the mechanical load. Bone remodeling takes place in the basic multicellular unit (BMU), which consists of osteoblasts, osteoclasts, and osteocytes within the bone-remodeling cavity. Physiological bone remodeling is the result of the harmonious coupling of bone resorption and bone formation. Bone resorption is mediated by osteoclastic activity, whereas bone formation by osteoblastic activity^6^. Osteoclasts are large, multinucleated cells that are produced by the fusion of mononuclear hematopoietic stem cells derived from the monocyte–macrophage lineage (osteoclastogenesis). Mature osteoclasts bind tightly to the bone and create a sealed microenvironment where they produce enzymes that affect the organic matrix, as well as acid that degrades the mineral component. Osteoblasts are mononuclear cells derived from mesenchymal stem cells (osteoblastogenesis) and they induce bone matrix formation, collagen synthesis, osteocalcin production, and mineralization. Osteoblasts ultimately become part of the mineralized matrix and they turn into osteocytes or bone-lining cells. Normal bone remodeling is the result of the balanced crosstalk among osteoclasts, osteoblasts, osteocytes, bone matrix, and immune cells that is reflected on the interrelated intracellular and extracellular molecular cascades and signaling molecules^6^. Herein, we describe the MM-induced deregulation of this process and provide rationale for future research.

### The emerging role of osteocytes

Osteocytes represent ~95% of all bone cells and play a key role in bone remodeling. They are embedded within the lacuno-canalicular network, however, they communicate with cells in the bone surface and bone marrow via cytoplasmic projections. Osteocytes express factors with paracrine action such as RANKL and sclerostin that regulate both the osteoblastic and osteoclastic activity^7^.

It has been shown that the number of viable osteocytes in MM patients is reduced compared to healthy controls and this is correlated with the extent of MM-induced disease^8^. The interaction between MM cells and osteocytes activates the Notch pathway reciprocally; regarding MM cells both Notch signaling and Notch receptor expression, particularly Notch3 and Notch4, are stimulated^9^. Subsequently, RANKL/OPG ratio is increased, osteoclasts precursors are recruited and activated leading to local bone resorption. In parallel, sclerostin inhibits Wnt signaling and, consequently, osteoblast differentiation; thus it further contributes to MM-induced bone loss^9, 10^. Interestingly, it has been recently reported that osteocyte apoptosis is crucial in modifying bone marrow local microenvironment and creating a pre-metastatic state that favors MM plasma cell homing and growth^11^. Further insight into this field might have implications in preventing bone dissemination of MM, as well as bone metastases of solid malignancies.

Regarding MM therapeutics, the proteasome inhibitor bortezomib has been shown to alter bone microenvironment in terms of osteocyte viability^12^. Patients treated with bortezomib had a significantly higher number of viable osteocytes compared to other MM patients. In vitro studies confirmed these results and also showed that bortezomib reduced autophagy-induced osteocytes death^12^.

### Cell to cell interactions

The crosstalk between MM plasma cells and bone marrow stromal cells (BMSCs) is critical for the induction of osteolytic disease. MM cells induce alterations in the bone marrow microenvironment and establish positive feedback vicious cycles that favor their survival^13^. BMSCs become deregulated and their differentiation into osteoblasts is impaired^14^. Both MM cells and BMSCs express cell adhesion molecules (CAMs) that mediate the mutual interactions. The very late antigen 4 (VLA-4) integrin system is of particular interest; MM cells express VLA-4 (α4β1 integrin) while BMSCs express VCAM-1, which is a ligand for VLA-4. Activation of the VLA-4 integrin system is critical for MM homing and development, as well as for MM-induced bone disease^15^. When it is disrupted, bone resorption is suppressed and MM cell proliferation is suspended^16^. The adhesive interactions also activate p38alpha mitogen-activated protein kinase (p38α-MAPK) that induces bone resorption and MM survival; preclinical inhibition of this pathway results in prevention of osteolytic disease and decreased tumor load^16^. Targeting cell-to-cell interactions is a promising therapeutic field that remains to be further investigated.

## Molecular pathways primarily implicated in increased osteoclast activity

### RANK/RANKL pathway

The receptor activator of nuclear factor (NF)-κB (RANK)/RANK ligand (RANKL) signaling pathway has been identified as a crucial regulatory system of bone remodeling. RANK is a transmembrane receptor that belongs to the tumor necrosis factor (TNF) superfamily and is expressed on the surface of osteoclast precursors. RANKL is a cytokine expressed as a membrane-bound protein by BMSCs of osteoblastic lineage and activated T-lymphocytes. The metalloprotease-disintegrin TNFα-converting enzyme mediates its cleavage into a soluble form^17^. The binding of RANKL to RANK leads to the fusion of osteoclast precursors into multinucleated cells that will ultimately become mature osteoclasts. The mature osteoclasts attach to the bone surface in order to become activated and the bone resorption process is initiated^18^. Osteoprotegerin (OPG) is a member of the TNF superfamily and is secreted by BMSCs and osteoblasts. It is a soluble decoy receptor for RANKL that inhibits osteoclastogenesis. Stimuli that favor bone destruction such as 1,25-dihydroxyvitamin D, PTH, PTHrP, glucocorticoids, cytokines (IL-1, IL-7, TNFα), and prostaglandin E2 exert a differential effect on the aforementioned molecules by increasing RANKL and decreasing OPG expression^17, 18^.

The deregulation of RANK/RANKL/OPG signaling pathway in MM bone disease has been well described^19^. MM cells interact with bone marrow microenvironment and activate molecular cascades that ultimately result in increased RANKL and decreased OPG expression^9, 12^. Apart from BMSCs, osteoblasts, and endothelial cells, RANKL is overexpressed in T-lymphocytes and MM cells^20^. Soluble RANKL produced by MM cells has been implicated in the development of bone destruction in MM patients^19, 20^. Furthermore, plasma cells also secrete PTHrP that in turn stimulates RANKL expression by osteoblasts and BMSCs via paracrine way^21^. In addition, MM cells express syndecan-1, an heparan sulfate proteoglycan, that binds to OPG. OPG is subsequently taken up by endocytosis in MM cell, where it is degraded^22^. It has been demonstrated that OPG levels are reduced in MM patients with osteolytic disease, while RANKL/OPG ratio has been proven to be an independent prognostic factor in a series of 121 newly diagnosed MM patients^23^.

The most promising agent targeting RANK/RANKL/OPG signaling pathway is the humanized monoclonal antibody denosumab. Due to its high affinity and specificity for RANKL, it can prevent RANK activation, osteoclastogenesis, and osteoclast activation. Recently, denosumab met the primary endpoint of a randomized, double blind, phase 3 study by demonstrating no inferiority compared with zolendronic acid in delaying time to first on-study SRE in patients with newly diagnosed MM. Furthermore, it was proven superior to zolendronic acid regarding time to first on-study SRE in a 15-month landmark analysis^24^. Interestingly, a PFS advantage was seen in denosumab arm; something that needs further investigation as this was an exploratory endpoint in the study^24^. Autologous stem cell transplantation (ASCT) normalizes the deregulated bone turnover and decreases RANKL/OPG ratio^25^, whereas bortezomib-based regimens lead to normalization of bone remodeling by reducing serum RANKL levels^26^. Restoration of RANKL/OPG ratio has been also achieved via inhibition of microRNA miR-21^27^. Syndecan-1, through which myeloma cells bind, internalize and degrade OPG, is another emerging therapeutic target that could alter MM microenvironment^28^.

### Notch pathway

Four transmembrane receptors (Notch 1–4) are included in the Notch family. When they bind to their ligands (Jagged 1,2 and Delta-like 1,3,4) expressed by adjacent cells, two proteolytic cleavages mediated by ADAM/TACE and γ-secretase complex are activated and the intracellular portion of Notch (ICN) is released. ICN translocates to the cell nucleus, induces transcriptional and epigenomic alterations, and ultimately activates HES-1 and C-MYC^29^.

Notch signaling pathway is actively implicated in MM-induced osteoclastogenesis^29^. Two ways of Notch pathway activation in MM have been described; MM cells express Notch 1, 2, 3 that can bind to their ligands presented either on the same cells (Jagged 1,2), namely homotypical interaction, or on adjacent BMSCs and malignant plasma cells (Jagged or Delta-like ligands), namely heterotypical interaction^30^. It has been reported that Jagged 2 deregulation in MM cells is an early phenomenon at the monoclonal gammopathy of undetermined significance (MGUS) stage, while the aberrant expression of Jagged 1 coincides with the transition from MGUS to MM^29^. The net effect of Notch activation is the production of the osteoclastogenic factor RANKL by MM cells. RANKL binds to the RANK receptor on the surface of osteoclasts precursors and, in turn, Notch2 expression and activation are induced. The Notch2 signaling cascade in pre-osteoclasts may be further stimulated by binding to Jagged ligands on neighboring MM cells. BMSCs also express Notch receptors that may be triggered by Jagged ligands of MM cells and subsequently increase RANKL production^29, 30^. Furthermore, there has been accumulating evidence indicating that Notch signaling pathway may be implicated in the establishment of a pre-metastatic milieu in the bone by triggering the expression of adhesion molecules, migratory chemokines, and angiogenetic factors and by disrupting the immune surveillance^9, 29^. Notch pathway inhibition induces MM cell apoptosis, enhances chemosensitivity, inhibits osteoclastogenesis, and MM cell migration to the bone marrow in the preclinical setting^31^. Thus, it is a promising field for future targeted drug development.

### Osteopontin

Osteopontin is a non-collagenous bone matrix glycoprotein secreted by osteoclasts and is implicated both in osteoclast activation and local angiogenesis^32^. In MM patients, osteopontin has been described as a dual marker of bone resorption and angiogenic activity; high osteopontin levels have been associated with extensive osteolytic lesions and advanced disease^33^. However, it has been reported that in a subset of MM patients with maf gene translocations, high osteopontin levels might exert a protective role on bone preservation by altering the crosstalk between MM cells and osteoclasts^34^.

### CCL-3 (MIP-1α)/CCL-20

Chemokine (C-C motif) ligand3 (CCL-3), previously known as macrophage inflammatory protein-1α (MIP-1α), is a chemokine secreted by MM plasma cells and plays an important role in the pathogenesis of MM-induced bone disease^35^. Chemokine (C-C motif) ligand 20 (CCL-20) is a chemokine involved in the Th17 pathway and is also implicated in MM osteolytic disease. CCL-20 and its receptor, CCR6, are overexpressed in the MM bone marrow niche and induce osteoclastogenesis. High levels of CCL-3/CCL-20 are detected in bone marrow and serum of MM patients and are positively correlated with the extent of bone disease and negatively associated with survival^36, 37^. In MM human cells with translocation t(4;14), upregulation of fibroblast growth factor receptor 3 induces CCL-3 expression; thus this may be a pathway through which the cytogenetic feature mediates its adverse effect^38^. The main receptors of CCL-3 are CCR1 and CCR5, they are expressed on BMSCs, osteoclasts, osteoblasts, and MM cells, and they may have differential effects on osteolytic bone disease and MM cell migration. Due to its chemotactic action, CCL-3 attracts osteoclast precursors and induces osteoclastogenesis, while it potentiates RANKL and IL-6 effects on osteoclasts^39^. Furthermore, CCL-3 inhibits osteoblast activity by downregulating RUNX2 and osterix, as well as osteoblast mineralization^40^. CCL-3 also promotes MM cell survival and is involved in the homing process of MM cells in the bone marrow niche^41^. In preclinical studies, inhibition of CCL-3 has been effective in preventing bone destruction by impeding osteoclast function and restoring osteoblast activity, whereas the tumor burden was reduced^37, 40^. CCR1 and CCR5 antagonists also inhibit osteoclastogenesis and MM cell adhesion to BMSCs^42^. Available biology data suggest that CCL-3 and its receptors are suitable targets for the development of novel drugs for osteoclast deregulation in myeloma patients.

### Activin A

Activin A is a member of the TGFβ superfamily that binds to the type II transmembrane serine/threonine kinase receptor (ActRIIA/B) that recruits and phosphorylates the type I receptor (ActRI, more commonly the activin receptor-like kinase 4 (ALK4) receptor) and a heterodimer is formed. In turn, ALK4 induces the activation of the Smad signaling cascade that results in the translocation of Smad2/3/4 complex in the nucleus, where it acts as a transcriptional factor. Apart from this canonical pathway, it has been reported that activin A is implicated in Smad-independent signaling pathways such as Akt/PI3K, MAPK/ERK, JNK, and WNT/β-catenin (non-canonical pathways)^43^. Activin A induces RANK expression and activates NF-κB pathway and, therefore promotes osteoclast differentiation^43^. Regarding bone involvement in solid malignancies, activin A has shown differential effects depending on the tumor microenvironment. Both bone marrow plasma and serum levels of activin A have been found increased in MM patients with lytic bone disease^36,44, 45^. Elevated circulating activin A levels have been associated with advanced MM features and adverse prognosis^44^. Increased activin A levels have also been correlated with elevated serum periostin in newly diagnosed MM patients^46^. It has been reported that the crosstalk between BMSCs and MM cells induces activin A secretion^45^. Furthermore, activin A inhibits BMP signaling pathway in MM cell lines. Preclinical data have shown inhibition of cancer-induced bone destruction via activin A blockade^47^. In a phase II clinical study, sotatercept (ACE-011), a ligand trap fusion receptor, showed anabolic efficacy in terms of bone mineral density and bone formation^48^. The rationale for combining lenalidomide, which does not reduce activin A levels, and an activin A inhibitor has been provided, and thus a relevant clinical trial is currently ongoing^44, 49^.

### Interleukins

Interleukin 3 (IL-3) is a bifunctional cytokine that stimulates osteoclast formation and inhibits osteoblast differentiation in preclinical MM models. IL-3 induces activin A production by bone marrow macrophages and, thus, it exerts its osteoclastogenic effect^50^. The osteoblast suppression is mainly mediated by the participation of CD45 + hematopoietic cells. Elevated IL-3 levels have been detected in the bone marrow plasma of MM patients compared with healthy controls^50^. It has been reported that the primary source of IL-3 are T-lymphocytes and not MM cells, underlying the role of immune regulation in MM bone disease.

Interleukin-6 (IL-6) is a multifunctional cytokine implicated in bone metabolism and is principally secreted by myeloid precursor cells. IL-6 enhances osteoclast differentiation, whereas it sustains MM cell survival. Additionally, it stimulates MM plasma cells to secrete vascular endothelial growth factor (VEGF) that further activates osteoclasts by binding to their surface receptors^32^. Serum IL-6 along with matrix metalloproteinase-9 levels have been correlated with bone turnover rate in MM patients. Restoration of bone remodeling has been provided with anti-IL-6 monoclonal antibodies and these results are investigated in clinical trials^51^. Interestingly, IL-6 levels have been reduced with DKK1 inhibition^52^. IL-6 also stimulates the PI3K/Akt/mTOR pathway, that in turn regulates IL-6, VEGF and osteopontin expression. A dual PI3K/mTOR inhibitor decreased osteoclast function and increased osteoblast formation in a preclinical setting and, therefore, it may be effective in bone disease in the clinical setting as well^5^.

Interleukin 17 (IL-17) is a pro-inflammatory cytokine that is mainly secreted by T-helper cells (Th17) along with other T-lymphocytes and natural killer (NK) cells. In preclinical MM models, IL-17 promotes osteoclast activation and induces osteolytic lesions^52^. An anti-IL-17A monoclonal antibody (AIN 457) has shown positive results in preclinical studies by inhibiting MM growth and survival and downregulating osteoclast cell number^53^.

### TNF superfamily

TNF-alpha (TNF-α) is a signaling cytokine that is elevated in MM patients and is involved in the pathogenesis of MM bone disease. TNF-α acts synergistically with RANKL and induces osteoclastogenesis^54^.

B cell-activating factor (BAFF) is a member of TNF superfamily that is secreted by BMSCs, osteoclasts, and MM cells. MM patients present with increased serum BAFF levels; BAFF binds to its receptor and the net downstream effect is the activation of NF-κB that promotes MM cell survival^55^. In a preclinical model, BAFF inactivation via a monoclonal antibody resulted in decreased osteoclastogenesis and improved survival^56^. Furthermore, BAFF expression had a prognostic value in myeloma patients; however, the addition of a monoclonal anti-BAFF antibody, tabalumab, to bortezomib plus dexamethasone did not increase PFS compared to placebo in a phase 2 study^57^.

### BTK and SDF-1α

Bruton’s tyrosine kinase (BTK) is a nonreceptor tyrosine kinase of the TEC family and is principally implicated in B cell receptor signaling pathway and osteoclast differentiation^58^. Stromal cell-derived factor-1α (SDF-1α) is a chemokine that mediates the migration and homing of myeloma cells and induces osteoclast activity after binding to CXC chemokine receptor type 4 (CXCR4)^59^. BTK is expressed in MM cells and it is positively correlated with CXCR4 expression^58^. Osteoclast precursors expressing BTK and CXCR4 migrate towards SDF-1α and, in turn, SDF-1α induces BTK activation in MM cells^58^. SDF-1α/CXCR4 pathway has been described as an important regulator of MM cell homing and it constitutes a promising therapeutic target^59^. BTK inhibition in preclinical models has reversed the adverse effects of osteoclast overactivation^58^. Ibrutinib, a selective BTK inhibitor, suppresses bone resorption by counteracting osteoclastic activity and inhibiting chemokine and cytokine secretion from BMSCs and osteoclasts^60^. Ibrutinib efficacy on MM and MM-induced bone disease is currently investigated in the clinical trial era.

### Annexin II

Annexin II (Annexin A2) is a calcium-dependent phospholipid-binding member of the annexin family and is expressed by endothelial cells, BMSCs, mononuclear macrophages, and malignant cells. Annexin II is upregulated in MM patients and in MM cell lines and stimulates MM cell adhesion and growth, angiogenesis, osteoblastic mineralization, osteoclastogenesis^5, 61^. Annexin II is secreted both by MM plasma cells and BMSCs, osteoblasts and osteoclasts^61^. In the clinical setting, elevated annexin II expression is associated with adverse prognostic features^61^. Annexin II constitutes a promising targetable factor affecting bone remodeling in MM.

### PU.1

PU.1 is a transcriptional factor that is essential for osteoclast formation, as well as for myeloid and B-lymphoid cells. Immunomodulatory anti-MM agents (IMiDS) downregulate PU.1 and, thus, they inhibit osteoclastogenesis and they are effective against MM-induced bone disease^62, 63^.

## Molecular pathways primarily implicated in suppressed osteoblast activity

### WNT pathway

Wingless and integration-1 (WNT) signaling pathway is another molecular cascade involved in the pathogenesis of bone disease in MM patients^17, 19^. When the WNT pathway is activated, the canonical WNT ligands bind to the WNT co-receptors LRP5/6 and one transmembrane receptor of the FDZ family. Subsequently, a complex that includes disheveled (DVL), Axin, FRAT1, and GSK-3β is created. In turn, β-catenin of the cytoplasm is able to translocate to the nucleus where it activates the T cell factor/lymphoid enhancer factor (TCF/LEF) transcription factors that ultimately result in gene expression favoring bone formation and impeding bone resorption. PTH binding to PTH1 receptor may also activate this pathway in the absence of WNT ligands. Furthermore, the non-canonical WNT–planar cell polarity (WNT–PCP) pathway is initiated by the formation of WNT ligand–receptor tyrosine kinase-like orphan receptor 2 (ROR2) or the receptor-like tyrosine kinase (RYK)–FZD–DVL complex and consists of three distinct cascades: the Disheveled-associated activator of morphogenesis 1 (DAAM1)–RHO–RHO-associated kinase (ROCK) pathway, the RAC–Jun kinase (JNK)–RUNX2 pathway and the WNT–Ca^2+^ pathway^64^. In preclinical MM models in vivo, it has been shown that increased WNT signaling affects the bone marrow microenvironment and prevents the development of myeloma bone disease^65^.

Interestingly, WNT–β-catenin pathway is also implicated in cell cycle promotion as well as in cell–cell interactions. Aberrant WNT signaling has been reported to contribute to the proliferation of MM cells, despite the absence of relevant identifiable mutations as in solid malignancies. This implies the role of bone marrow niche in sustaining WNT signaling and the increased sensitivity of MM plasma cells to autocrine and paracrine WNT signals^66^. Importantly, it has been suggested that members of the WNT family favor the migration and invasion of myeloma plasma cells through the non-canonical WNT pathway as well^67^. This pathway is also implicated in the adhesion-mediated drug resistance of MM cells^68^. Inhibition of the deregulated WNT pathway is a promising target with encouraging results in preclinical studies^66^. WNT pathway inhibitors such as sclerostin, Dickkopf-1, and sFRP-2/3 seem to be elevated and induce bone resorption by preventing β-catenin translocation to the nucleus^69^.

### Sclerostin

Sclerostin is a cysteine knot-containing protein, produced by osteocytes, resulting from the transcription of the SOST gene. Sclerostin induces the apoptosis of mature osteoblasts by activating the caspase pathway and inhibits osteoblast-driven bone formation^70^. Sclerostin antagonizes the activation of the canonical WNT pathway by binding to the extracellular domain of LRP5/6 transmembrane receptors that are found on osteoblast-lineage cells^70^. Thus, WNT ligands cannot bind to LRP5/6, DVL–Axin–FRAT1–GSK-3β complex is not formed and GSK-3β phosphorylates β-catenin. B-catenin subsequently undergoes proteasomal degradation via ubiquitination. Inside the nucleus, TCF/LEF transcription factors are repressed by Groucho and gene expression promoting bone formation is suspended^64^. Furthermore, sclerostin, alone or in conjunction with noggin, prevents type I and type II bone morphogenetic proteins (BMPs) from binding to their receptors and, thus, the BMP-mediated mineralization in osteoblasts is downregulated^71^. It has been shown that sclerostin is secreted by MM cells derived from the bone marrow of MM patients^72^. Sclerostin suppresses bone formation by inhibiting the osteoblastogenesis and the mineralization process, while it stimulates osteoclastogenesis by augmenting RANKL/OPG ratio^73^. Interestingly, elevated circulating sclerostin levels have been found in MM patients compared to patients with MGUS^74^. MM patients presented with fracture at diagnosis had higher levels of circulating sclerostin, as well as those with International Staging System (ISS)-3 disease^74^. High levels of sclerostin have been described even in the plateau phase of disease course^75^. Elevated circulating sclerostin has been proposed as an adverse prognostic factor in MM patients^74^. Sclerostin is a potential therapeutic target of monoclonal antibodies. Romosozumab, a humanized anti-sclerostin antibody, has been proven effective in restoring bone remodeling in benign bone disorders^4^. The rationale for a similar approach in MM has been provided by preclinical studies, especially by combining anti-sclerostin antibodies with antitumor drugs such as proteasome inhibitors^10^.

### Dickkopf-1

Dickkopf-1 (DKK1) is a member of the DKK family that antagonizes the WNT pathway and plays an important role in osteoblastogenesis and skeletal development^69^. DKK1 binds to LRP5/6 in combination with the Kremen1/2 transmembrane proteins and forms a complex that induces the internalization of LRP; thus, the activation of the canonical WNT/β-catenin pathway is inhibited^76^. DKK1 has emerged as a key modulator of bone disease in MM^69^. It inhibits osteoblastogenesis since it prevents BMSCs from differentiating into mature osteoblasts by suspending the autocrine WNT signaling that is necessary for the BMP-2-mediated osteoblast differentiation^77^. In turn, the undifferentiated BMSCs secrete interleukin-6 (IL-6) that stimulates the proliferation of MM plasma cells secreting DKK1^78^. DKK1 acts synergistically with sclerostin and results in osteoblast dysfunction^64^. Furthermore, DKK1 deregulates the RANKL and OPG production that is mediated by WNT pathway in osteoblasts resulting in an increase of RANKL/OPG ratio; thus, osteoclastogenesis is indirectly stimulated^79^. Elevated serum and bone marrow DKK1 levels are associated with the presence of lytic bone lesions in MM patients^36^. A positive correlation between DKK1 levels and the amount of osteolytic lesions has been also described^77^. Genomic studies have shown that increased expression of DKK1 gene is associated with myeloma-induced bone disease^80^. Importantly, high DKK1 expression is a predictor of early SRE incidence even in patients on bisphosphonate treatment^81^. Successful anti-myeloma treatment, including bortezomib-based regimens, immunomodulatory (IMiDS) agents or ASCT, is associated with normalization of bone remodeling that is mediated, at least in part, by reduced serum DKK1 levels^26, 82^. These results have provided the rationale for developing DKK1 neutralizing antibodies, such as BHQ880 that induces osteoblast differentiation and inhibits myeloma cell growth via alterations in the bone marrow microenvironment^83^. In a phase II clinical trial including patients with high risk smoldering MM, it demonstrated increased bone anabolic activity^84^. Interestingly, targeting DKK1 may also have important implications in the immunotherapy of MM patients, as active vaccination of murine MM models with DKK1-DNA vaccine was effective in preventing MM development and reducing tumor burden in mice with established MM^85^.

### Periostin

Periostin, also known as osteoblast-specific factor, is a disulfide-linked cell adhesion protein that belongs to the fasciclin family and it is produced by BMSCs. It is expressed in the periosteum as a response to mechanical load and it is implicated in cell migration of bone-remodeling cells. Periostin is expressed during osteoclast differentiation in vitro, as well as in the early phases of osteoblast differentiation, whereas it decreases during mineralization stage. For this reason, it has been suggested as a potential marker of new bone formation^86^. Periostin is a multifaceted molecule; it acts as a structural component of the bone matrix, while it is also implicated in the WNT-signaling pathway and it activates the integrin–AKT–FAK–β-catenin pathway^87^. Periostin is implicated in several forms of cancer and MM by promoting tumor growth and metastasis. Both newly diagnosed and relapsed patients present with elevated levels of periostin in bone marrow plasma and in serum compared to SMM, MGUS, and healthy controls. High periostin levels are also associated with extensive bone lytic lesions, bone fractures and advanced disease characteristics^46^. Interestingly, monoclonal antibodies targeting periostin have emerged with promising results in preclinical models of breast and ovarian cancer^88^; nevertheless, their role in MM pathogenesis remains to be investigated.

### RUNX2, GFI1, and IL-7

Runt-related transcription factor 2/core-binding factor Runt domain subunit 1 (RUNX2/CBFA1) is part of the non-canonical WNT-signaling pathway and constitutes a critical regulator of osteoblastogenesis. MM cells inhibit RUNX2 activity in BMSCs and osteoblast precursor cells and, therefore, they impede osteoblast differentiation^14^. Indeed, MM patients with osteolytic disease have reduced number of RUNX2-positive osteoblasts and stromal cells in their bone marrow biopsies compared with those without bone lesions^14^. Recently, cysteine-rich 61 (CYR61/CCN1) protein, that is secreted in the bone marrow microenvironment, has been identified as a stimulator of osteoblast differentiation by upregulating RUNX2 in MM preclinical studies^89^. However, it has been shown that MM cells also overexpress RUNX2 and higher levels are associated with advanced disease characteristics and poor prognosis. RUNX2 induces the Akt/β-catenin/survivin pathway along with the transcriptional activation of a gene panel that facilitate the homing of MM cells into the bone niche^90^. RUNX2 is also a major regulator of osteopontin, as previously discussed.

Growth factor independence-1 (GFI1) is a transcriptional repressor that binds to RUNX2 and decreases its expression. BMSCs derived either from MM patients or MM-bearing mice have shown increased levels of GFI1^91^. Importantly, anti-TNF-α and anti-IL-7 antibodies suppress GFI1 activity, whereas siRNA knockdown of GFI1 restores RUNX2 levels^91^. Therefore, GFI1 is a promising target in MM bone disease. Moreover, GFI1 recruits histone deacetylases and other epigenetic modifiers to the RUNX2 promoter; inhibition of these molecules has emerged as a feasible way of reversing the long-term suppression of osteoblast activity in MM^92^.

Interleukin 7 (IL-7) downregulates RUNX2 transcriptional activity and, thus, it inhibits osteoblast differentiation^14^. Moreover, IL-7 stimulates T-lymphocytes to secrete RANKL^20^. Increased IL-7 levels have been found in bone marrow plasma of MM patients^20^. IL-7 is also involved in the RUNX2-mediated osteoblast suppression by inducing GFI1^91^. Overall, targeting RUNX2, GFI1, and IL-7 might have encouraging results in overcoming MM-induced bone destruction.

### TGFβ and BMPs

Transforming growth factor β (TGFβ) is produced in an inactive form by osteocytes and osteoblasts in bone matrix and it is activated by osteoclasts during bone resorption^93^. Similar to periostin, TGFβ has been suggested as a potential marker of bone formation^86^. Deregulation of TGFβ pathway is implicated in cancer-induced bone disease. TGFβ induces arrest of terminal BMSCs differentiation in MM microenvironment. Importantly, TGFβ inhibition restores terminal osteoblast differentiation and suppresses MM cell growth^93^. Thus, targeting TGFβ could be a significant therapeutic potential in the future.

BMPs are included in the TGFβ superfamily and act through Smad-dependent and Smad-independent pathways. BMP-2 induces osteoblastogenesis and promotes bone formation^94^. MM cells overexpress hepatocyte growth factor (HGF) and Pim-2 kinase; both of them have been described as negative regulators of BMP-mediated osteoblast differentiation^95^.

### TNF superfamily

Apart from its role in promoting osteoclastogenesis, TNF-α inhibits osteoblast precursor recruitment from progenitor cells and suppresses RUNX2 and its transcriptional co-activator, TAZ, as well as osterix; resulting to osteoblast differentiation^91^.

LIGHT is another member of TNF superfamily that binds to the membrane-bound TNF signaling receptors, HVEM and lymphotoxin beta receptor (LTβR), and activates the TNF-receptor-associated-factor cascade that ultimately alters gene expression^96^. In patients with MM bone disease, LIGHT is secreted by immune cells including CD14+monocytes, CD8+ T cells, and neutrophils. LIGHT decreases osteoblastogenesis by inhibiting the formation of osteoblast precursors, osteocalcin and collagen I as well as by inducing sclerostin expression by monocytes. In parallel, LIGHT favors osteoclast differentiation by acting synergistically with RANKL and activating the Akt, NFκB and JNK signaling pathways^96^. Anti-LIGHT monoclonal antibodies might restore the deregulated bone metabolism.

### EphrinB2/EphB4 signaling pathway

The Eph receptors are tyrosine kinase receptors that are activated by ligands called ephrins (Eph receptor-interacting proteins) and their bidirectional counteracting function plays a substantial role in bone metabolism. EphrinB2 is expressed in osteoclasts and is induced by PTH, while EphB4 is expressed in osteoblasts and BMSCs^97^. EphrinB2/EphB4 binding results in two signaling cascades; the forward signaling that favors osteoblast differentiation by downregulating RhoA and the reverse signaling that inhibits osteoclast differentiation by suppressing Fos and Nfatc1 transcription^97^. Both EphrinB2 and EphB4 expression is decreased in BMSCs of MM patients. Decreased WNT signaling in MM may reduce EphB4 expression by osteoblasts. Chimeric EphrinB2-Fc-activated EphB4 in BMSCs and EphB4-Fc-stimulated EphrinB2 activation in osteoclasts. The administration of these agents in murine MM models resulted in enhancement of bone formation indices^98^. Thus, targeting EphrinB2/EphB4 signaling pathway could reverse the pathogenesis of bone disease in MM.

### Adiponectin

Adiponectin constitutes an adipocyte-derived hormone that is also expressed by osteoblasts and BMSCs. Both osteoblasts and osteoclasts express adiponectin receptors, and adiponectin exerts its effect on bone remodeling through autocrine/paracrine and endocrine pathways. The direct pathway by circulating adiponectin is inhibitory, while all the others are stimulatory^99^. In a murine MM model, adiponectin deficiency was associated with bone disease and increased tumor load, whereas pharmacologic stimulation of adiponectin secretion prevented MM-induced bone lesions and resulted in prolongation of survival^100^. The crosstalk between adipocytes and MM cells remains currently under investigation.

## Conclusion

**Fig. 1:**
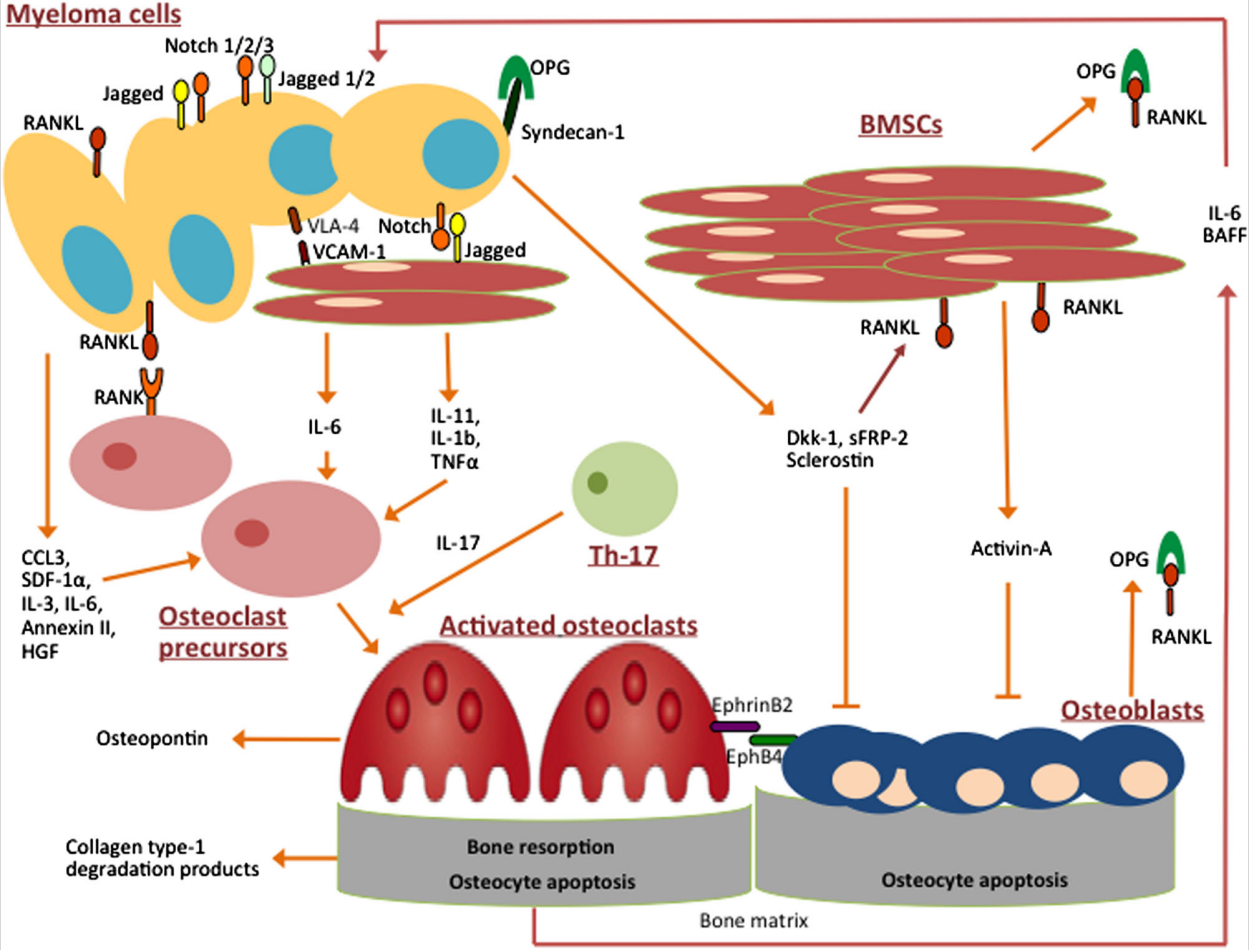
Schematic overview of myeloma-related bone disease The intercellular interactions between BMSCs and MM cells along with the involvement of immune cells, such as Th17 cells, induce cytokine release (IL-1b, IL-3, IL-6, IL-11, IL-17) and secretion of pro-osteoclastogenic factors such as TNF-α, CCL-3, SDF-1α, and annexin II in the bone marrow microenvironment. These cytokines promote increased osteoclast activity and inhibit osteoblastogenesis. Adhesion molecules such as VCAM-1 on BMSCs and VLA-4 on MM cells mediate cell-to-cell contact. Notch, expressed by MM cells, binds to Jagged, expressed by neighboring MM cells and BMSCs, and activate intracellular cascades favoring RANKL production. MM cells also enhance the apoptosis of osteocytes that also release RANKL. RANKL binds directly to RANK on osteoclast precursors and promotes osteoclastogenesis. Syndecan-1 on MM cells binds and inactivates OPG, the RANKL soluble decoy receptor. Osteoclasts also produce factors sustaining MM cell growth and survival, including IL-6 and BAFF. Furthermore, MM cells produce soluble factors that inhibit osteoblastogenesis such as DKK1, sFRP-2, and sclerostin. Activin-A secreted by BMSCs also impedes osteoblast production, while at the same time activates osteoclasts. EphB4 on osteoblasts and BMSCs binds to EphrinB2 on osteoclasts and results in bidirectional signaling that ultimately induces osteoclastogenesis and impedes osteoblastogenesis. All these interactions lead to increased osteoclast activity, diminished osteoblast function, increased bone resorption, bone destruction and development of osteolytic lesions, and/or pathological fractures

Pathogenesis of bone disease in MM constitutes a multifaceted entity that includes several intracellular and intercellular signaling pathways (Fig. 1). Molecular pathways such as RANK/RANKL/OPG, Notch, Wnt, RUNX2, EphrinB2/EphB4, and TNF pathway, as well as signaling molecules including DKK1, sclerostin, periostin, osteopontin, GFI1, BMPs, TGFβ, activin A, annexin II, adiponectin, BTK, SDF1a, chemokines, and interleukins, are at the spotlight of current MM research (Table 1). Despite the current evidence regarding the uncoupling of the remodeling process, the crosstalk among the regulators of bone turnover is only partially understood. However, targeting key factors implicated in MM-induced bone disease has been fruitful; denosumab has shown no inferiority compared to zolendronic acid in a phase III clinical trial. However, we need not only to target osteoclasts but also to enhance osteoblast function and target osteocytes as well. Therefore, preclinical research along with clinical trials investigating bone-related outcomes are deemed indispensable to improve the management of bone disease in MM patients.

**Table 1:** Summary of the currently molecular targets and therapeutic implications in myeloma-related bone disease

Molecular target	Use in MM/therapeutic implication
*Increased osteoclast activity*	
RANK/RANKL pathway	Denosumab (anti-RANKL moAb). Phase 3 clinical trial completed: denosumab was not inferior to zoledronic acid; possibly superior regarding PFS^24^
	RANKL/OPG is reduced by ASCT^25^
	RANKL is reduced by bortezomib-based regimens^26^
Syndecan-1	Preclinical setting^27^
Notch pathway	Preclinical setting
Osteopontin	Preclinical setting
CCL-3 (MIP-1α) / CCL-20	Preclinical setting^37,40, 42^
Activin A	Sotatercept (ACE-011) (ligand trap fusion receptor). Phase 2 clinical trial completed: sotatercept increased BMD in MM patients who received MPT^48^
	Lenalidomide+Activin A inhibitor. Phase 1 clinical trial^49^
Interleukin-6	Anti-IL-6 moAbAnti-MM activity in clinical trials^51^
Interleukins 3 and 17	Preclinical setting^52, 53^
PI3K/Akt/mTOR pathway	Preclinical setting^5^
TNF-α	Preclinical setting
BAFF	Tabalumab (anti-BAFF moAb). Negative results in a phase 2 clinical trial^57^
BTK and SDF-1α	Ibrutinib (selective BTK inhibitor). Ongoing clinical trials
Annexin II	Preclinical setting
PU.1	Downregulated by IMiDs^62, 63^
*Suppressed osteoblast activity*	
WNT pathway	Preclinical setting^65, 66^
Sclerostin	Preclinical setting in MM^10^
	Romosozumab, an anti-sclerostin moAb, for benign bone disorders^4^
Dickkopf-1 (DKK1)	BHQ880 (DKK1 neutralizing Ab). Increased bone anabolic activity in a phase 2 clinical trial^84^
Periostin	Preclinical setting^88^
RUNX2, GFI1 and IL-7	Preclinical setting^91, 92^
TGFβ and BMPs	Preclinical setting
TNF-α and LIGHT	Preclinical setting
EphrinB2/EphB4 signaling pathway	Preclinical setting^98^
Adiponectin	Preclinical setting^100^

*RANK/RANKL* receptor activator of nuclear factor (NF)-κB (RANK)/RANK ligand, *moAb* monocloncal antibody, *ASCT* autologous stem cell transplant, *BMD* bone mineral density, *CCL* chemokine (C-C motif) ligand, *MIP-1α* macrophage inflammatory protein-1α, *IL* interleukin, *PI3K/Akt/mTOR* phosphatidylinositol-3-kinase (PI3K)/Akt and the mammalian target of rapamycin, *TNF* tumor necrosis factor, *BTK* Bruton’s tyrosine kinase, *SDF-1α* stromal cell-derived factor-1α, *WNT* wingless and integration-1, *MM* multiple myeloma, *MPT* melphalan, thalidomide, prednisone, *RUNX2* runt-related transcription factor 2, *GFI1* growth factor independence-1, *TGFβ* transforming growth factor β, *BMPs* bone morphogenetic proteins, *PFS* progression-free survival
